# Bioactivity and Bone Cell Formation with Poly-ε-Caprolactone/Bioceramic 3D Porous Scaffolds

**DOI:** 10.3390/polym13162718

**Published:** 2021-08-13

**Authors:** Po-Kai Juan, Fang-Yu Fan, Wei-Chun Lin, Pei-Bang Liao, Chiung-Fang Huang, Yung-Kang Shen, Muhammad Ruslin, Chen-Han Lee

**Affiliations:** 1 Division of Prosthodontics, Department of Dentistry, Taipei Medical University Hospital, Taipei 11031, Taiwan; jordan6412@yahoo.com.tw (P.-K.J.); peibang@gmail.com (P.-B.L.); 2 School of Dental Technology, College of Oral Medicine, Taipei Medical University, Taipei 11031, Taiwan; fish884027@tmu.edu.tw (F.-Y.F.); tukust94114wenny@gmail.com (W.-C.L.); chiung0102@tmu.edu.tw (C.-F.H.); 3 Division of Family and Operative Dentistry, Department of Dentistry, Taipei Medical University Hospital, Taipei 11031, Taiwan; 4 Department of Oral and Maxillofacial Surgery, Faculty of Dentistry, Hasanuddin University, Makassar 90245, Indonesia; m.ruslin@vumc.nl; 5 Taiwan Society of Blood Biomaterials, New Taipei City 221, Taiwan; b210103011@tmu.edu.tw; 6 School of Dentistry, College of Oral Medicine, Taipei Medical University, Taipei 110, Taiwan

**Keywords:** 3D composite scaffold, improved solvent-casting/particulate-leaching, bioceramic, physical and degradation properties, biocompatibility

## Abstract

This study applied poly-ε-caprolactone (PCL), a biomedical ceramic powder as an additive (nano-hydroxyapatite (nHA) or β-tricalcium diphosphate (β-TCP)), and sodium chloride (NaCl) and ammonium bicarbonate ((NH_4_)HCO_3_) as porogens; these stuffs were used as scaffold materials. An improved solvent-casting/particulate-leaching method was utilized to fabricate 3D porous scaffolds. In this study we examined the physical properties (elastic modulus, porosity, and contact angle) and degradation properties (weight loss and pH value) of the 3D porous scaffolds. Both nHA and β-TCP improved the mechanical properties (elastic modulus) of the 3D porous scaffolds. The elastic modulus (0.15~1.865 GPa) of the various composite scaffolds matched that of human cancellous bone (0.1~4.5 GPa). Osteoblast-like (MG63) cells were cultured, a microculture tetrazolium test (MTT) was conducted and alkaline phosphatase (ALP) activity of the 3D porous scaffolds was determined. Experimental results indicated that both nHA and β-TCP powder improved the hydrophilic properties of the scaffolds. The degradation rate of the scaffolds was accelerated by adding nHA or β-TCP. The MTT and ALP activity tests indicated that the scaffolds with a high ratio of nHA or β-TCP had excellent properties of in vitro biocompatibility (cell attachment and proliferation).

## 1. Introduction

Tissue engineering techniques used in bone reconstruction and regeneration often require a temporary porous scaffold, which modulates the growth of cells migrating from surrounding tissue or of cells seeded inside the porous structure of the scaffold. Porosity and pore interconnectivity within the scaffold are critical for cell adhesion, proliferation, and the diffusion of nutrients and oxygen throughout such 3D constructs. The scaffold should also provide appropriate mechanical properties to support the regeneration of damaged tissues [1].

A solvent-casting/particulate-leaching method was developed to prepare highly porous poly (L-lactic acid) (PLLA) membranes. Their properties are independent of the salt type and are only affected by the weight fraction and particulate size of the salt [2]. Porosity is an important consideration when designing scaffolds for tissue engineering, since a proper morphology is needed to promote cell growth and the proliferation and transportation of nutrients into poly-ε-caprolactone (PCL)/hydroxyapatite (HA) scaffolds [3].

Pore interconnectivity is another important factor in scaffolds used for tissue engineering. A layer-by-layer technique was used to fabricate PCL/chitosan (CS) scaffolds, with salt used as the porogen [4]. Another technique used NaCl to improve pore interconnectivity in PCL/HA composite scaffolds. Experimental results revealed that the scaffolds had high interconnectivity and good cell adhesion and proliferation [5]. Another group added ammonium bicarbonate to scaffolds, which formed pores as the ammonium bicarbonate vaporized. This allowed water to seep into the scaffold causing the NaCl particles to leach out. However, PCL has poor mechanical properties [6]. Therefore, HA particles were added to PCL scaffolds to enhance the mechanical properties and promote osteoconductivity [7]. Researchers who added HA found a significantly increased compressive modulus. Their experimental results revealed higher osteoblast differentiation in PCL/HA scaffolds than in PCL scaffolds [8]. A novel PCL/nano-fluoridated HA (PCL-FHA100) nanocomposite scaffold was developed. Results showed that the compressive strength of the blended scaffolds increased with an increase in the weight ratio of FHA100 and decreased with an increase in porosity [9]. In vitro degradation behaviors were investigated using three-dimensional (3D) porous/CS scaffolds. Results revealed that the degradation rate of a PCL/CS scaffold was faster than that of a pure PCL scaffold [10]. A PCL/HA scaffold with a high HA content degraded faster than one with a lower HA content. All PCL/nano (n)-HA composites revealed decreased compressive strengths and decreased moduli as the severity of degradation increased, especially in scaffolds with high HA contents [11].

A centrifugation method was developed for fabricating cylindrical PCL scaffolds. In vitro cell interactions were investigated using chondrocytes, osteoblasts, and fibroblasts. In vivo tissue interactions were examined on scaffolds in a rabbit model, and bone formation was enhanced by large pore sizes (290~310 μm) [12]. Other research summarized the effect of pore sizes on tissue regeneration and reported that a pore size of 100~350 μm is conducive to bone generation [13]. PCL/nHA scaffolds (with a pore size of 400 μm and fixed to a titanium bone plate) were implanted into rats and rabbits in an in vivo study. Results indicated that PCL/nHA scaffolds were effective in promoting bone regeneration [14]. Processing/structure/property relationships of multi-scale PCL and PCL-HA composite scaffolds prepared via a combined gas-foaming/porogen-leaching technique were assessed. The multi-scale scaffolds allowed the 3D osteogenic differentiation of MG63 cells [15]. Porous scaffolds created by thermally induced phase separation consisted of aggregated microparticles of a PCL homopolymer and PCL-HA nanocomposite. The prepared scaffolds had porosities in the range of 80~92%, pore sizes of up to 600 μm, and high pore interconnectivity [16]. PCL/HA and PCL/halloysitr nanotube (HNT) composite scaffolds were prepared by co-extrusion and gas foaming. Compressive properties of PCL/HNT scaffolds were higher than those of PCL/HA scaffolds. Human mesenchymal stem cells (hMSCs) proliferated and differentiated best on 5% HA scaffolds followed by 1% HNT scaffolds and 5% HNT scaffolds [17]. A novel calcium phosphate (CaP)/PCL scaffold was fabricated by a solvent-casting/particle-leaching process. A porosity gradient was clearly obtained, whereas the gradation in the phase composition was less pronounced [18].

Microporous membranes for guided tissue regeneration based on PCL and on two different functionalized PCL and relative HA nanocomposites were realized by solvent/non-solvent phase inversion. A PCL-dimethylaminoethyl acrylate (DMAEA)/HA combination was most promising in terms of cell adhesion, proliferation and differentiation [19]. PCL-HA30 nanocomposite micro-scaffolds were used to induce hMSC differentiation without the addition of osteogenic growth factors. Moreover, the presence of an inorganic cue (HA) on the micro-particle surface positively affected differentiation towards an osteogenic lineage [20]. PCL/HA composite blends exhibited high loading contents of HA. Application of an ultrasonic wave during the extrusion process aided in providing sufficient processability and flowability of PCL/HA blends with up to 30 wt.% of HA. This ultrasound-assisted extrusion process has potential to replace the solution-mixing technique [21]. HA/PCL composite scaffolds with a bimodal architecture of small and large macropores were prepared by a two-step scCO_2_ process and were coated with potato dextrose agar (PDA) to incorporate two different growth factors (GFs). This resulted in faster release of vascular endothelial growth factor (VEGF) compared to bone morphogenetic protein (BMP)-2. While BMP-2 secretion took place during the entire healing process, VEGF expression occurred in the early healing stage [22]. The NIPS-based 3D plotting technique is useful in producing porous PCL/HA composite scaffolds with a controlled macro/micro-porous structure, high mechanical properties, and good bioactivity. With an increase in the HA content, the mechanical properties (i.e., compressive yield strength) and apatite-forming ability significantly increased [23]. A novel 3D electrospun PCL/HA nanofiber (NF) composite scaffold was developed to mimic the natural bone matrix, and it was utilized in combination with BMP-2 for improved in vitro osteogenesis and in vitro new bone formation [24]. An indirect 3D printing method was applied to manufacture porous scaffolds to facilitate the design of desired scaffold shapes and control the formation of macro-and micro-structures. The composite made with a PLA/PCL ratio of 70/30 containing 35% HA was more suitable when considering porosity and the degradation rate [25]. The 3D-printed PCL/HA scaffolds were found to be cytocompatible and capable of osteogenic differentiation and antimicrobial activity in vitro [26]. Aligned and non-aligned PLLA/PCL fibers and PLLA/PCL/HA scaffolds were produced by an electrospin-electrospray process. Aggregates of nanophase HA improved the mechanical properties of these blends, by acting as a reinforcement and enhancing the responses of these constructs to tensile stress [27]. PCL/HA hybrid microspheres consisting of two substances, PCL and HA, were produced by a spray-precipitation technique. Results demonstrated that the proliferation and osteogenic differentiation of hPDCs were improved by the addition of HA to PCL microspheres [28]. Porous PCL/nHA composite scaffolds were fabricated by a modified melt-molding/leaching technique using a combination of salt particulate and polyethylene glycol (PEG) as co-porogens. This scaffold prepared from NaCl/PEG presented many macropores with interconnectivity and showed high strength and good bioactivity [29]. Porous scaffolds were produced by a thermal crosslinking of PCL diacrylate in the presence of HA and a particulate leaching technique with sodium chloride as the water-soluble porogen. Levels of alkaline phosphatase (ALP) activity were found to be higher for PCL/HA network scaffolds than for PCL network scaffolds [30]. An in situ sol gel method was introduced to prepare HA/PCL nanocomposites. Young’s modulus and the ultimate tensile strength of PCL was enhanced by the addition of 20 wt.% HA nanoparticles (NPs) [31]. Interconnected porous PLA scaffolds were developed via a SSE/water-soluble porogen leaching approach. The presence of PEG not only generated desirable processability of PLA/NaCl, but also facilitated the occurrence of interconnected pores. The compressive modulus that varied from 85.7 to 207.4 MPa fell in the range of a normal modulus of human trabecular bone (50~250 MPa) [32]. Porous PCL/HA composite scaffolds with a controlled macro/micro-porous structure were produced using NIPS-based 3D plotting. The mechanical properties (i.e., ultimate tensile strength and compressive yield strength) and apatite-forming ability significantly increased with an increase in the HA content [33]. A solvothermal process was employed to prepare nano-rod HA (20%)-reinforced PCL composite scaffolds. The prepared scaffolds could be promising for non-load-bearing bone regeneration because of their good mechanical properties and ability to promote apatite growth [34]. A successful synthesis of HA based on abalone mussel shells with a molar ratio of Ca/P of 1.67 was presented. Overall the cell metabolic activity and morphology of the HA + HCB 30 wt.% scaffolds showed that they were able to facilitate attachment of MC3T3E1 cells on their surface [35]. Porous scaffolds were fabricated with PCL/β-tricalcium phosphate (BTCP at 10, 20, and 50 wt.%) biocomposites by a freeze-drying method. The porosity of these scaffolds was 80~85%. The adhesion and proliferation of bone marrow-derived MSCs seeded onto PCL/BTCP scaffolds were enhanced compared to those on PCL [36].

Controlled and sustained release of vitamin C from tricalcium phosphate (TCP) scaffolds was observed in the presence of a PCL coating. The sustained release of vitamin C from PCL-coated TCP scaffolds enhanced the proliferation and differentiation of osteoblast cells [37]. In a 3D-printing system, PCL-BO and PCL-DCB exhibited greater ability for osteoinduction than synthetic materials such as PCL-HA or PCL-TCP. Doping 3D-printed PCL scaffolds with DCB or BO might better support in vivo bone healing compared to TCP- or HA-doped grafts [38]. Brain tissue reactions to PCL and PCL-TCP scaffolds were investigated by inflammatory response staining (IBA-1). Results suggested brain tissue compatibility with PCL-TCP scaffolds [39]. The feasibility of 3D-printed PCL/β-TCP scaffolds was evaluated in large defects of the canine mandible. Scaffolds incorporating heterogeneous pore sizes exhibited rapid bone ingrowth, and those with additional wing structures had more-stable screw fixation [40]. As FDM allowed the fabrication of tailor-made bioresorbable rhBMP-2/mPCL-TCP/collagen scaffolds with fully interconnected channel networks and adequate mechanical properties, the delivery of rhBMP-2 from mPCL-TCP/collagen scaffolds represents a promising advance for clinical applications in craniofacial surgery [41]. Various PTF/CH scaffolds were fabricated using a simple coating process, and enhanced mechanical and cellular behaviors of hierarchical scaffolds (PTF) consisting of PCL/β-TCP struts and electrospun PCL nanofibers were observed. Cell viability reasonably improved with PTF/CH scaffolds relative to PTF scaffolds and PTF/CH_5_ scaffolds displayed the best cell viability [42]. PCL/TCP scaffolds with two various fiber laydown patterns were coated with HA and gelatin. The groups with coated scaffolds showed lower bone formation and lower biomechanical properties within the defect compared to uncoated scaffolds [43]. Pliable and strong scaffolds were obtained by modulating PDLLA/PCL ratios. β-TCP not only enhanced the strength but also improved the scaffold’s hydrophilicity. Scaffolds with pore sizes in the range 200~500 μm met the minimum requirements as a bone scaffold and the porosity exceeded 80% [44]. PCL/β-TCP/βdECM/BMP scaffolds were produced by printing βdECM containing rhBMP-2 between PCL/β-TCP lines using a 3D printer. βdECM stably carried rhBMP-2 and was found to enhance cell adhesion and promote osteogenic differentiation [45]. Different PCL/CH/gelatin samples were fabricated by adding various amounts of β-TCP. MG63 cell attachment, proliferation, and morphology, as well as type I collagen gene expression, degradation rate, swelling and mechanical properties were optimized in a sample containing 3% β-TCP [46]. PCL-based fibrous nanocomposite membranes embedded with spherical α-TCP nanopowder were engineered as a drug carrier to use as hemocompatible and bioactive substrates for bone tissue engineering. The 1 wt.% α-TCP nanopowder (PCL-1α membrane) resulted in enhanced mechanical properties, bioactivity, and hydrophilicity of PCL-based membranes [47]. Influences of the composition and porosity of FDM 3D-printed PCL/β-TCP constructs were studied for bone tissue engineering. That study provided guidance for designing PCL/β-TCP constructs and provided the feasibility of single-piece constructs integrating multiple porosities and composite compositions [48]. The research extruded β-TCP/PCL filaments at different ratios, sterilized samples with clinically available E-beam irradiation, and implanted them in subcutaneous sites for 24 weeks. Adding TCP to PCL significantly increased composite degradation, but an increasing TCP content in the composite did not accelerate degradation [49]. Preclinical assessments of bone-replacing materials are numerous, but papers that examined clinical circumstances of bone graft applications and consumer market acceptance are rare. One research study evaluated dental adoption of bone grafts in accordance with the perception of dentists in Brazil. The authors interviewed 183 professional dentists. The most frequently mentioned compositions were ceramics, followed by composites. Autogenous and xenogeneic grafts were more frequently used than alloplastic or allogeneic ones, and ceramics were the most frequently used composition [50].

Traditional layer-by-layer 3D printing cannot easily fabricate complex shapes on products. 3D free-form surface printing enables omni-directional printing of brackets to produce complex shapes (such as spirals). In their study, by introducing an in situ inorganic NP precipitation process to a 3D freeform printing system with a two-step crosslinking strategy, the authors successfully fabricated HAc-Alg/CaP nanocomposite hydrogel scaffolds with various mineral contents and good structural integrity [51].

In this study, nHA or β-TCP was added to the PCL matrix to construct composite scaffolds. An improved solvent-casting/particulate-leaching method was used to fabricate 3D porous scaffolds. Effects of various ratios of nHA or β-TCP on the physical properties, degradability, and biocompatibility of the composite scaffolds were investigated. This study also discusses the results of cell culture using pure PCL and composite scaffolds.

## 2. Experimental

### 2.1. Scaffold Preparation

**Figure 1:**
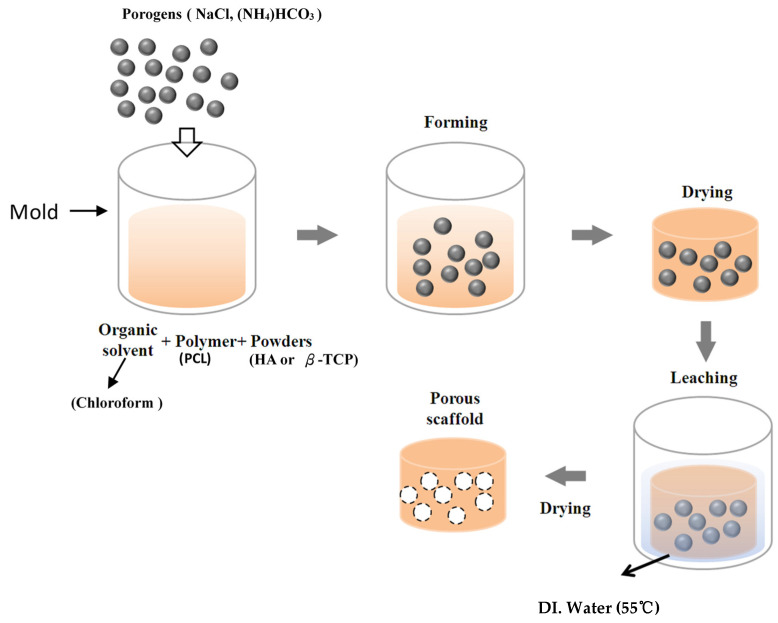
Flowchart of the fabrication of the scaffolds.

Porous scaffolds were fabricated by an improved solvent-casting/particulate-leaching method using PCL (440744, Sigma-Aldrich, St. Louis, MO, USA) as the scaffold material. The porogens were NaCl (Sigma-Aldrich, St. Louis, MO, USA) ranging 250~400 μm in size and ammonium bicarbonate ((NH_4_)HCO_3_). A flowchart of the scaffold fabrication procedure is shown in Figure 1. Table 1 lists the preparation and characteristics of various scaffolds. First, PCL (Mn: 80,000) was dissolved in chloroform at 1:10 wt/vol at room temperature for 12 h. NaCl and (NH_4_)HCO_3_ mixed with nHA (Sigma-Aldrich, St. Louis, MO, USA) or β-TCP (Sigma-Aldrich, St. Louis, MO, USA) were added to the PCL matrix and stirred (The traditional solvent-casting/particulate-leaching method does not use (NH_4_)HCO_3_). The mixed PCL solution (PCL+HA or β-TCP) was poured into a dish and dried at room temperature for 90 min. Residual chloroform was removed by vacuum drying at 100 μmHg and 25 °C for 2 h. After fabrication, the scaffolds were immersed in deionized (DI) water at 55 °C to leach out the NaCl and (NH_4_)HCO_3_ for 1 h and vacuum-dried for 12 h. The completed scaffold was then stored for further use. The fabrication time of each porous scaffold was 2 days. The fabrication time of the composite scaffold using the improved solvent-casting/particulate-leaching method (2 days) was shorter than that employing the solvent-casting/particulate-leaching method (7 days). DI water could not seep into the scaffolds, so the NaCl was unable to be leached out with the solvent-casting/particulate-leaching method. The authors needed to heat the water to 55 °C with the improved solvent-casting/particulate-leaching method.

### 2.2. Physical Properties

The porosity of the scaffold was measured using Archimedes’ principle [52]. The porosity of a scaffold immersed in a bottle full of ethanol was determined by

(1) Porosity(%)=(w1−w3−ws)ρe(w1−w3)ρe+wsρs

where *w_s_* is the weight of the scaffold, *w*_1_ is the bottle weight when filled with ethanol, *w*_2_ is the bottle weight when filled with ethanol and the scaffold, *w*_3_ is the bottle weight after removing the ethanol-saturated scaffold from *w_2_*, and *ρ_e_* and *ρ_s_* are the ethanol density and scaffold density, respectively. The scaffold was then further measured by a mercury porosimeter (Belpore MP, Microtrac Retsch, Germany) to determine the pore size. Mercury was filled to gradually shrink the pores by applying external pressure. Surface morphologies and pore sizes of the PCL, PCL/nHA, PCL/β-TCP, and PCL/nHA/β-TCP scaffolds were observed by scanning electron microscopy (SEM, Hitachi S-240, Tokyo, Japan), after a thin gold (Au) layer had been deposited on the scaffold using a sputter coater under a vacuum.

A Hung Ta 9102 machine was employed to evaluate the compressive properties (elastic modulus) of the PCL, PCL/nHA, PCL/β-TCP, and PCL/nHA/β-TCP scaffolds with a load cell of 2000 N at a compression rate of 1 mm/min. Five samples (4 mm in diameter and 10 mm in height) were processed, and results were averaged per group. The stress was estimated by σ=FA=FπD24, where σ is the stress, F is the load (N), and *A* is the area (mm^2^). The elastic modulus (E) was determined by the slope of the stress-strain diagram. 

A contact angle meter (DIGIDROP DGD-DI) was applied to measure the contact angles of the PCL, PCL/nHA, PCL/β-TCP, and PCL/nHA/β-TCP scaffolds. Contact angles of composite scaffolds are used to discuss their surface properties (hydrophilic and hydrophobic). Five points were measured on each scaffold. DI water (0.5 µL) was dropped onto a scaffold’s surface. The three states of a solid/gas/liquid affected the liquid drop stability, and computer-controlled photography (25 frames/s) was used to capture images and convert the image files.

### 2.3. Degradation Test

Degradation tests were performed to measure the degradation rate of the scaffolds in the human body. Scaffolds were trimmed to 10 × 10 × 2 mm^3^ and placed in separate vials containing 3 mL of phosphate-buffered saline (PBS, Gibco, Grand Island, NY, USA). Scaffold specimens were degraded by incubation at 37 °C in PBS for up to 15 weeks without changing the medium. Each week, three samples in each group were washed five times in DI water, air-dried overnight, and vacuum-dried for at least 24 h. The weight loss and pH variation of the scaffolds were determined after incubation.

The pH value of PBS was measured with a pH meter (model 710-2, Waltham, WA, USA, Orion) in the degradation test. The weight loss (W_loss_) of each scaffold specimen was calculated using the following formula:

(2) $$W_{\mathrm{loss}}=\frac{W_{\mathrm{init}}-W_{\deg}}{W_{\mathrm{init}}}$$

where W_init_ and W_deg_ are the respective weights of the scaffold specimen before and after degradation.

### 2.4. Cell Culture

Cell adhesion and growth behaviors were compared among the PCL, PCL/nHA, PCL/β-TCP, and PCL/nHA/β-TCP scaffolds after seeding MG63 osteoblast-like cells in the scaffolds. MG63 osteoblast-like cells were cultured in a tissue culture flask at 37 °C in a 5% CO_2_ atmospheric environment. Dulbecco’s modified Eagle medium (DMEM, Hyclone, Logan, UT, USA) was used as the culture medium. Cells were treated with 0.25% trypsin-EDTA before being cultured in the scaffolds. Scaffolds were cut into sample sizes of 10 × 10 × 2 mm^3^. Samples were sterilized by washing twice with 75% or 95% ethanol and then rinsing in PBS three times for 15 min to remove any residual ethanol. Each sample was transferred to a 24-well tissue culture plate and seeded with 0.5 mL of the cell suspension at a concentration of 10^4^ cells/mL. Samples and plates were stored in an incubator for 7 days. The medium was refreshed every 3 days during this period. After 1, 4, and 7 days of cell culture, samples were removed [53]. Extracts for indirect tests were collected from scaffolds under standard conditions (ISO 10993-5). Scaffolds were immersed in complete culture medium for 120 h, at 37 °C without agitation. The ratio between the sample surface and the volume of the extraction vehicle was 2 cm^2^/mL. A negative control was represented by the extraction vehicle with no material, while the positive control was the same medium containing a 0.1% phenol solution. Pure extracts (100%) and 50% and 20% dilutions (prepared using complete culture medium) were added to cells, and seeded in 96-well plates for 24 h previously. After 3 days of incubation, the viability and metabolic activity of cells were tested by a 3-(4,5-dimethilthiazol-2-yl)-2,5-diphenyltetrazolium bromide (MTT) assay. Spectrophotometric absorbance was measured at 570~690 nm using an enzyme-linked immunosorbent assay (ELISA) reader. As a result, the optical density (OD) of cells was obtained [54].

In cytotoxicity tests, the viability and metabolic activity of cells, previously incubated with material extracts, were checked by the MTT assay. MTT into the mitochondria [54].

Cell proliferation in the sample scaffolds was assessed by a 5-bromo-4-chloro-indolyl-phosphatase (BCIP)-nitroblue tetrazolium (NBT) assay (Gibco) of ALP activity and cellular ALP production. This method indirectly measures ALP activity based on a chromogenic reaction initiated by the cleavage of the phosphate group of BCIP by ALP present in cells [55]. Protons produced by the reaction reduce NBT to an insoluble purple precipitate. This assay was applied to assess ALP production of MG63 cells in composite scaffolds after 1, 4, and 7 days of cell culture. After removing the culture medium, 200 μL of the BCIP/NBT solution was added to each well. Cells in the well were then incubated for 2 h at 37 °C. Next, MG63 cells were observed by inverted light microscopy, and insoluble purple precipitates were solubilized with 210 μL of sodium dodecylsulfate (SDS) in 10% HCl. After overnight incubation, the OD of the solubilized product was measured in a microliter plate reader. The mean absorbance and standard deviation (SD) were determined at 595 nm. Three independent experiments were performed, and each test was performed in triplicate wells for each treatment variable.

### 2.5. Statistical Analysis

In this study, measured data were subjected to statistical analyses. For any given experiment, each data point represents the mean ± standard deviation (SD) of six individual experiments. A *t*-test was used to determine the significance between groups in the MTT and ALP assays. Statistical significance is indicated by * *p* < 0.05.

## 3. Results and Discussion

### 3.1. Physical Properties of the Scaffolds

**Figure 2:**
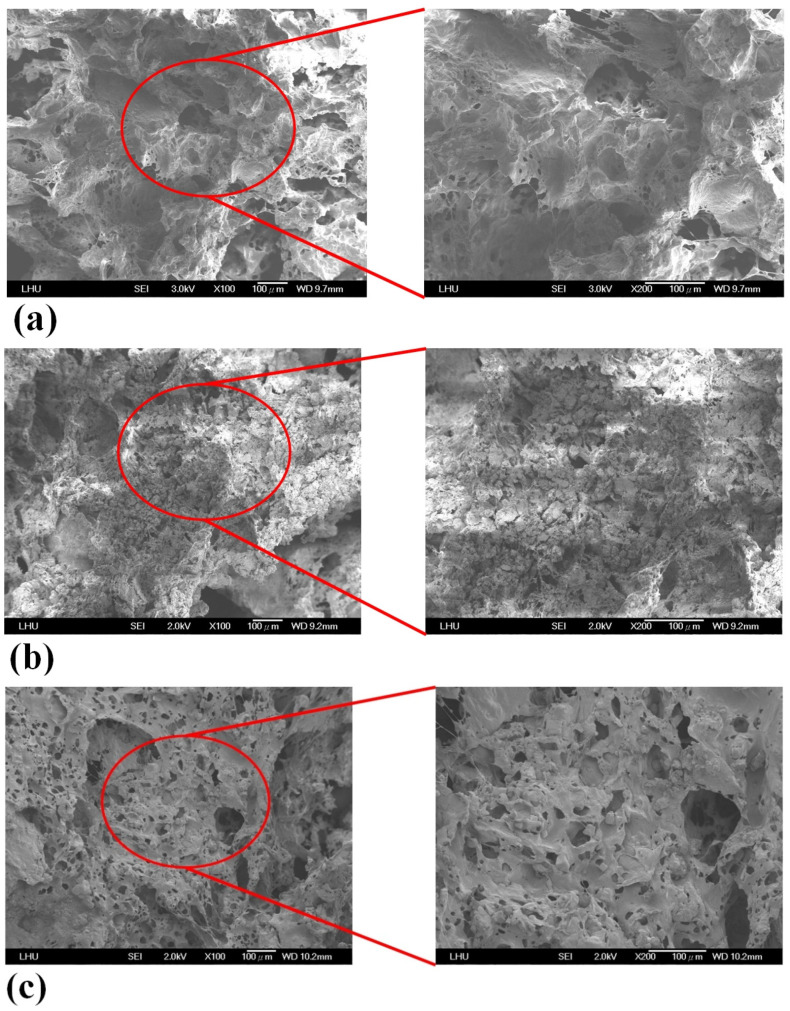

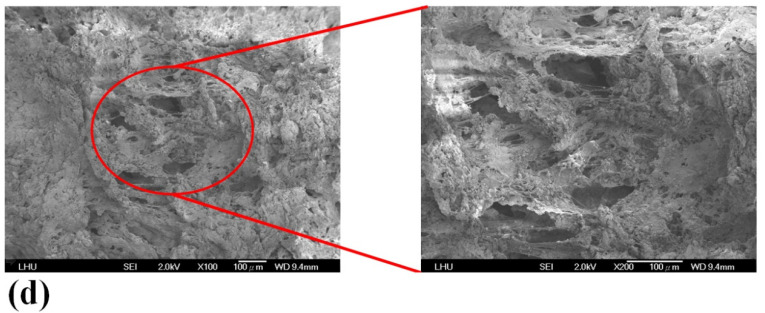
SEM images of (**a**) poly-ε-caprolactone (PCL), (**b**) PCL/nHA-3, (**c**) PCL/β-TCP-3, (**d**) PCL/nHA/β-TCP-6 scaffolds.

Scaffold samples were observed by SEM. Figure 2 reveals pore morphologies and surface structures of the PCL, PCL/nHA, PCL/β-TCP, and PCL/nHA/β-TCP scaffolds. Pore sizes varied from 110 to 350 μm in the four types of scaffolds. The pore size of the PCL scaffold was the largest. The reason was that those NPs (nHA and β-TCP) were smaller than the pores, so they easily filled in the pores of the scaffold caused by Van der Waals forces. Table 1 indicates the porosity of the 3D composite scaffolds with various ceramic materials. nHA and β-TCP particles were evenly exposed and dispersed on the scaffold’s surfaces as shown in Figure 2. This figure also compares the porosities of the PCL, PCL/nHA, PCL/β-TCP, and PCL/nHA/β-TCP scaffolds. Scaffolds with high ratios of ceramic powder (whether nHA, β-TCP, or nHA/β-TCP) had lower porosities compared to PCL scaffolds. The reason is that as the ceramic ratio increased, because the sizes of ceramic particles are on a nanometer scale, the Van der Waals effect caused ceramic particles to aggregate together. The NPs easily filled the scaffolds’ pores. The porosity of the 3D composite scaffolds was independent of the ceramic particle used (whether nHA, β-TCP, or nHA/β-TCP). The PCL scaffold had a maximum value of pore porosity because the nanometer-sized ceramic particles did not plug up the pores. Presumably, highly porous structures enable sufficient interaction of cells with a scaffold. Large pores in a scaffold increase diffusion of nutrients and gas but also reduce cell attachment. In contrast, small pores in a scaffold increase cell attachment but have poor nutrient and gas delivery [56]. According to the literature, a pore size of 100~350 μm in a scaffold is needed for bone regeneration and osteoconduction [11,12]. Cell growth and new tissue formation depend on the scaffold’s porosity, pore size, and material. An interconnecting pore network in a scaffold is also essential for tissue growth.

Table 1 shows values of the measured elastic modulus of the PCL, PCL/nHA, PCL/β-TCP, and PCL/nHA/β-TCP scaffolds. Clearly, the mechanical properties of the scaffolds improved after adding ceramic powder. Values of the elastic modulus of the various scaffolds were in the decreasing order of PCL/nHA/β-TCP scaffold > PCL/nHA scaffold > PCL/β-TCP scaffold > PCL scaffold. The compressive strength and hardness of the ceramic material (nHA or β-TCP) were greater than those of the PCL material. The elastic modulus of the PCL scaffold was smaller than that of the PCL scaffold with ceramic particles (nHA, β-TCP, or nHA/β-TCP). The elastic modulus of the composite scaffold (PCL with ceramic particles) increased as the ratio of ceramic particles increased [57]. The elastic modulus of the PCL/nHA scaffolds was larger than that of PCL/β-TCP scaffolds when the same ratio of particles was added [53]. The reason is that the compressive strength and hardness of β-TCP were smaller than those of nHA. The results also indicated that the PCL/nHA/β-TCP scaffold had a larger value of the elastic modulus due to this scaffold possessing a larger ratio of nHA particles. The elastic modulus of human cancellous bone is in the range 0.1–4.5 GPa [58,59], and the different composite scaffolds in this study were all in this range.

Table 1 reveals that the contact angles of the samples provided by the PCL/nHA, PCL/β-TCP, and PCL/nHA/β-TCP scaffolds had better hydrophilic properties compared to the PCL scaffold. The improved hydrophilic properties were achieved by the high specific area of the NPs [14,53,57,60]. The nHA and β-TCP NPs were more hydrophilic. Results also revealed that the contact angle of the composite scaffold with a high ratio of ceramic powder (nHA, β-TCP, or nHA/β-TCP) was smaller than that of the composite scaffold with a low ratio of ceramic powder. The contact angle of the PCL/nHA scaffold was a little smaller than that of the PCL/β-TCP scaffold which had the same ratio of nHA or β-TCP.

### 3.2. Degradation Test of the Scaffolds

**Figure 3:**
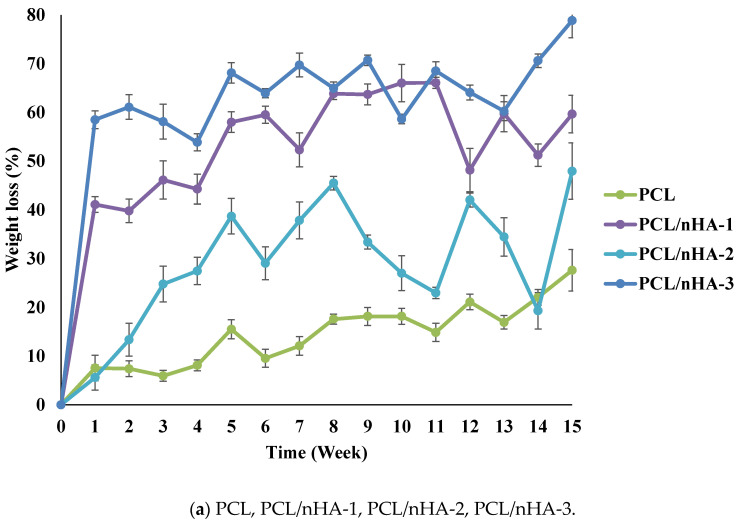

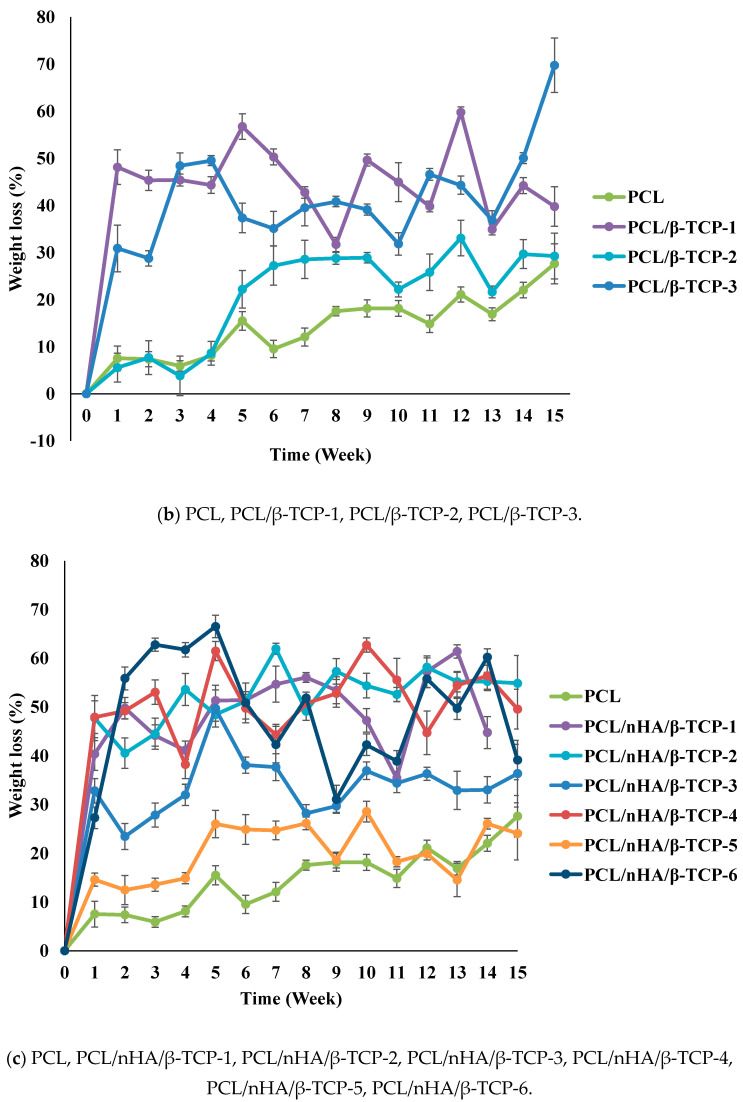
Time histories of the relative weight loss of poly-ε-caprolactone (PCL)/nHA, PCL/β-TCP, and PCL/nHA/β-TCP scaffold samples.

Variations in the degradation rates of the scaffolds were studied by performing a degradation test for 15 weeks. The weight loss and pH variation of the scaffolds were measured. Figure 3 illustrates the time histories of weight loss of the PCL, PCL/nHA, PCL/β-TCP, and PCL/nHA/β-TCP scaffolds. Results revealed that the PCL scaffolds exhibited a slow degradation rate. The maximum weight loss of PCL scaffolds was approximately 27.6%. Additionally, weights of the PCL/nHA, PCL/β-TCP, and PCL/nHA/β-TCP scaffolds had obviously decreased after 1 week. The weights of the PCL/nHA-3 and PCL/β-TCP-3 scaffolds eventually decreased to approximately 60% after 15 weeks. Weight loss and degradation of the scaffolds obviously decreased as the ratio of the ceramic powder increased. The weight loss of the PCL/nHA, PCL/β-TCP, and PCL/nHA/β-TCP scaffolds had increased by at least 20% by the end of the 15th week. The rank of weight loss was as follow: PCL/nHA-2 < PCL/nHA-1 < PCL/nHA-3. The PCL/nHA-2 scaffold had the smallest weight loss. The rank of weight loss revealed that the PCL/β-TCP-2< PCL/β-TCP-1< PCL/β-TCP-3. The PCL/β-TCP-2 scaffold exhibited the smallest weight loss. The rank of weight loss was as follow: PCL/nHA/β-TCP-6 < PCL/nHA/β-TCP-5 < PCL/nHA/β-TCP-3 < PCL/nHA/β-TCP-1 < PCL/nHA/β-TCP-4 < PCL/nHA/β-TCP-2. The smallest weight loss was for the PCL/nHA/β-TCP-6 scafflid. The low degradation rate of the PCL scaffold was expected because it is highly crystalline and hydrophobic. The degradation rate of the composite scaffolds (PCL mixed with ceramic powders) inevitably increased because they were highly hydrophilic.

**Figure 4:**
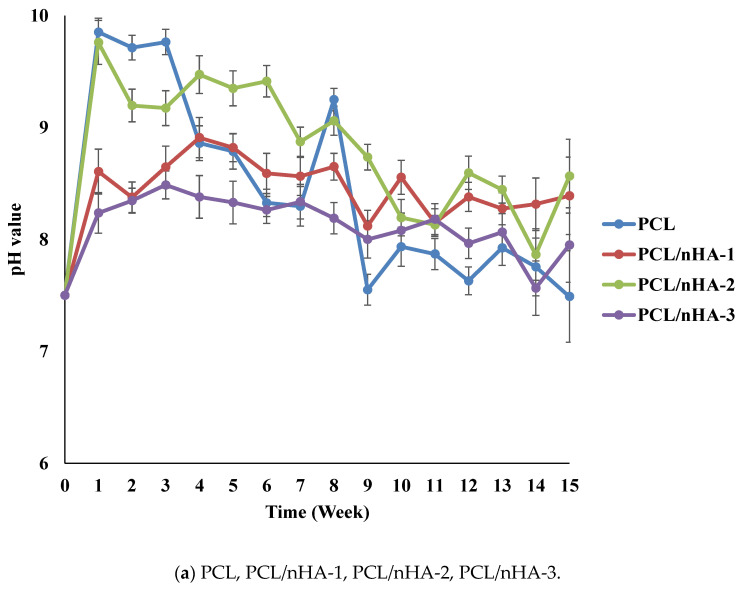

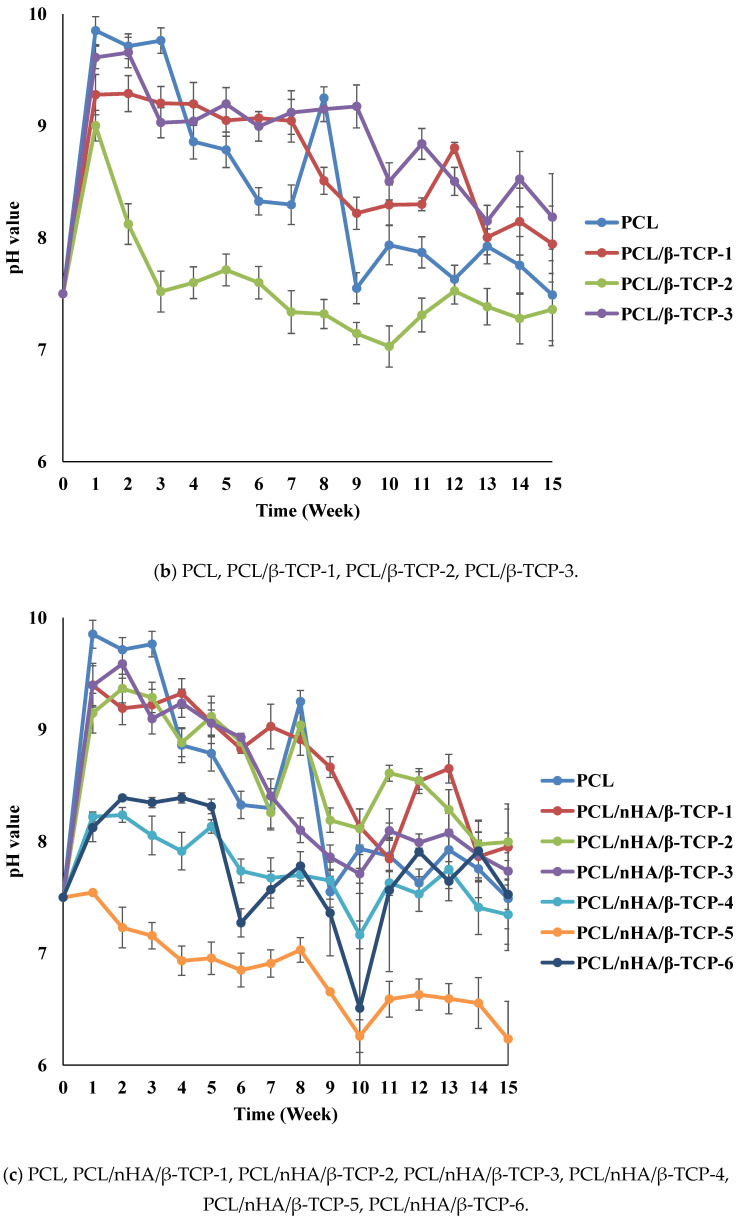
Time histories of pH values of poly-ε-caprolactone (PCL), PCL/nHA, PCL/β-TCP, and PCL/nHA/β-TCP scaffold samples.

The pH variations of the PCL and composite scaffolds were determined during the degradation test. Figure 4 indicates differences in degradation rates between the PCL and composite scaffolds. Since (NH_4_)HCO_3_ is alkaline, pH values of the scaffolds increased in the first week. The pH values of the PCL and composite scaffolds decreased because the PCL scaffolds released carboxyl according to the degradation test. The rank of pH values was PCL/nHA-2 > PCL/nHA-1 > PCL/nHA-3. The PCL/nHA-2 scaffold had the highest pH value. The rank of pH values was PCL/β-TCP-2 > PCL/β-TCP-1 > PCL/β-TCP-3. The PCL/β-TCP-2 scaffold had the highest pH value. The rank of pH values was PCL/nHA/β-TCP-1 > PCL/nHA/β-TCP-2 > PCL/nHA/β-TCP-3 > PCL/nHA/β-TCP-6 > PCL/nHA/β-TCP-4 > PCL/nHA/β-TCP-5. The largest pH value was for the PCL/nHA/β-TCP-1 scaffold. The pH values of the composite scaffolds remained in the range 6.25~7.0 during the 15-week experimental period. Such stability was expected since nHA and β-TCP are weakly basic and were released during degradation. These experimental results indicated that the ceramic powder and (NH_4_)HCO_3_ could neutralize the acid. Since the pH values of the scaffolds remained neutral, inflammation would likely not be induced in the human body.

### 3.3. Cell Culture

**Figure 5:**
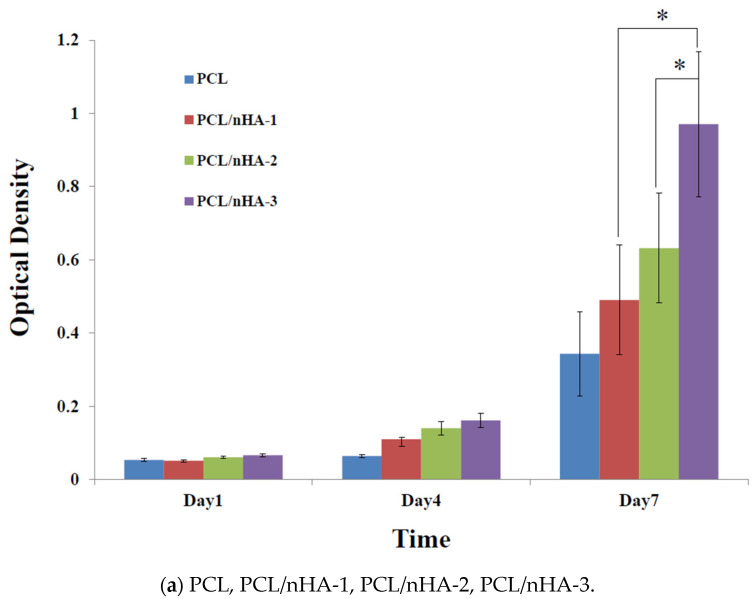

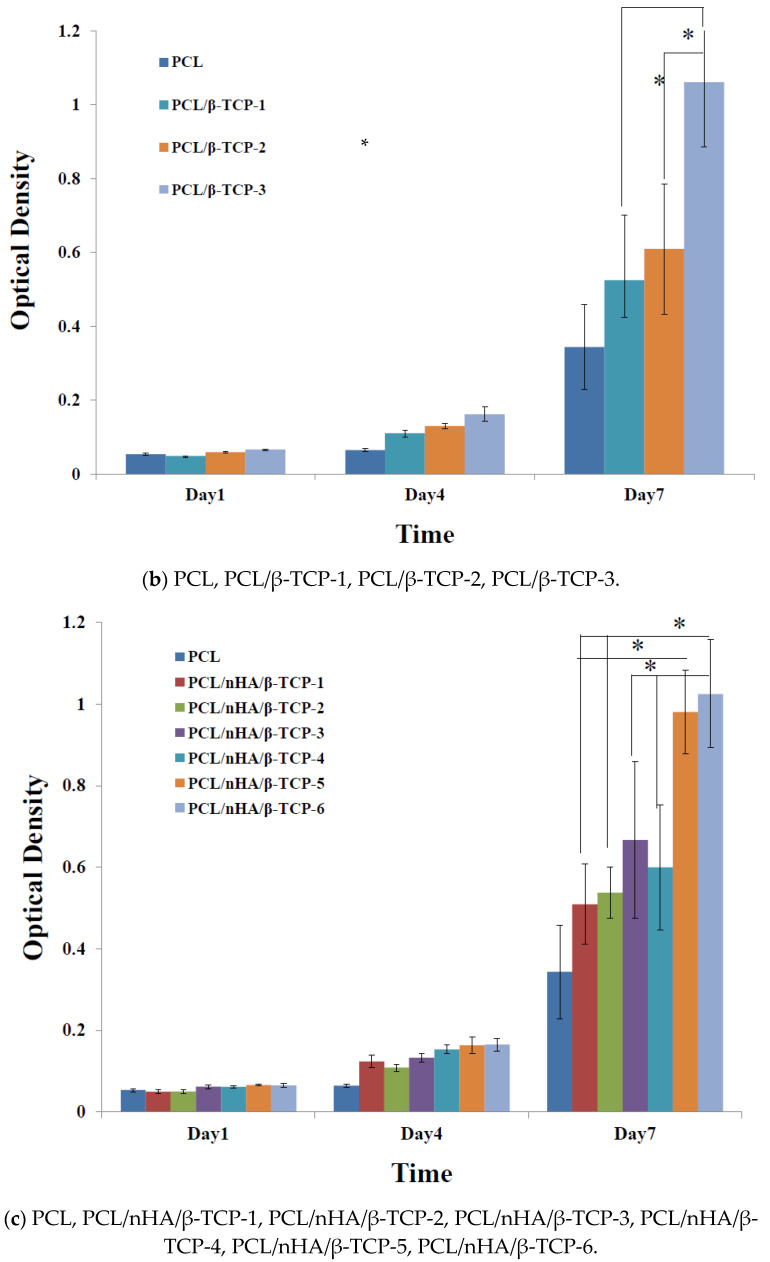
MTT assay of MG63 cells in poly-ε-caprolactone (PCL), PCL/nHA, PCL/β-TCP, and PCL/nHA/β-TCP scaffold samples. (* *p* < 0.05).

Proliferation of MG63 osteoblast-like cells cultured in the PCL, PCL/nHA, PCL/β-TCP, and PCL/nHA/β-TCP scaffolds was measured using an MTT assay, as shown in Figure 5. Cell proliferation increased as the cell culture time increased. After the first day of cell culture, OD values of cell cultures in the PCL and composite scaffolds did not significantly differ. After 7 days of cell culture, OD values of the PCL/nHA-3, PCL/β-TCP-3, PCL/nHA/β-TCP-5, and PCL/nHA/β-TCP-6 scaffolds were significantly greater than those of the other scaffolds. Therefore, NPs have an important role in supporting cell proliferation in scaffolds [61,62,63,64]. OD values of cells in the PCL/nHA, PCL/β-TCP, and PCL/nHA/β-TCP scaffolds were higher than those in PCL scaffolds, and this situation indicated that nHA and β-TCP contributed to cell growth. OD values of PCL scaffolds were lower than those of other scaffolds, because the HA or β-TCP powder improved the hydrophilic properties, which improved cell proliferation in the scaffolds with large amounts of these two powders [63,64]. The following was a separate comparison of the OD values of 3D composite scaffolds containing nHA, /β-TCP, nHA/β-TCP. The rank of OD values was PCL/nHA-3 > PCL/nHA-2 > PCL/nHA-1. The PCL/nHA-3 scaffold had the largest OD value. The rank of pH values was PCL/β-TCP-3 > PCL/β-TCP-2 > PCL/β-TCP-1. The PCL/β-TCP-3 scaffold had the greatest OD value. The rank of OD valuea was PCL/nHA/β-TCP-6 > PCL/nHA/β-TCP-5 > PCL/nHA/β-TCP-4 > PCL/nHA/β-TCP-3 > PCL/nHA/β-TCP-2 > PCL/nHA/β-TCP-1. The largest pH value was situated at PCL/nHA/β-TCP-6 scaffold.

**Figure 6:**
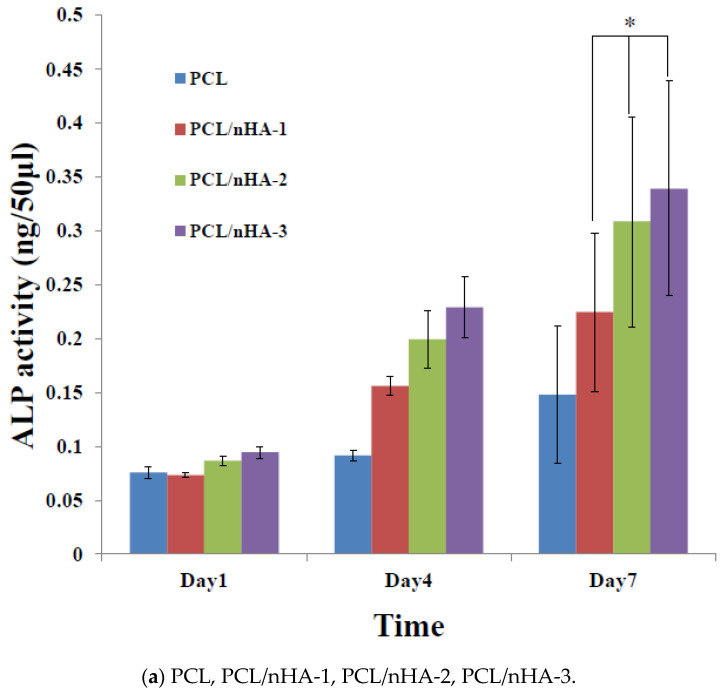

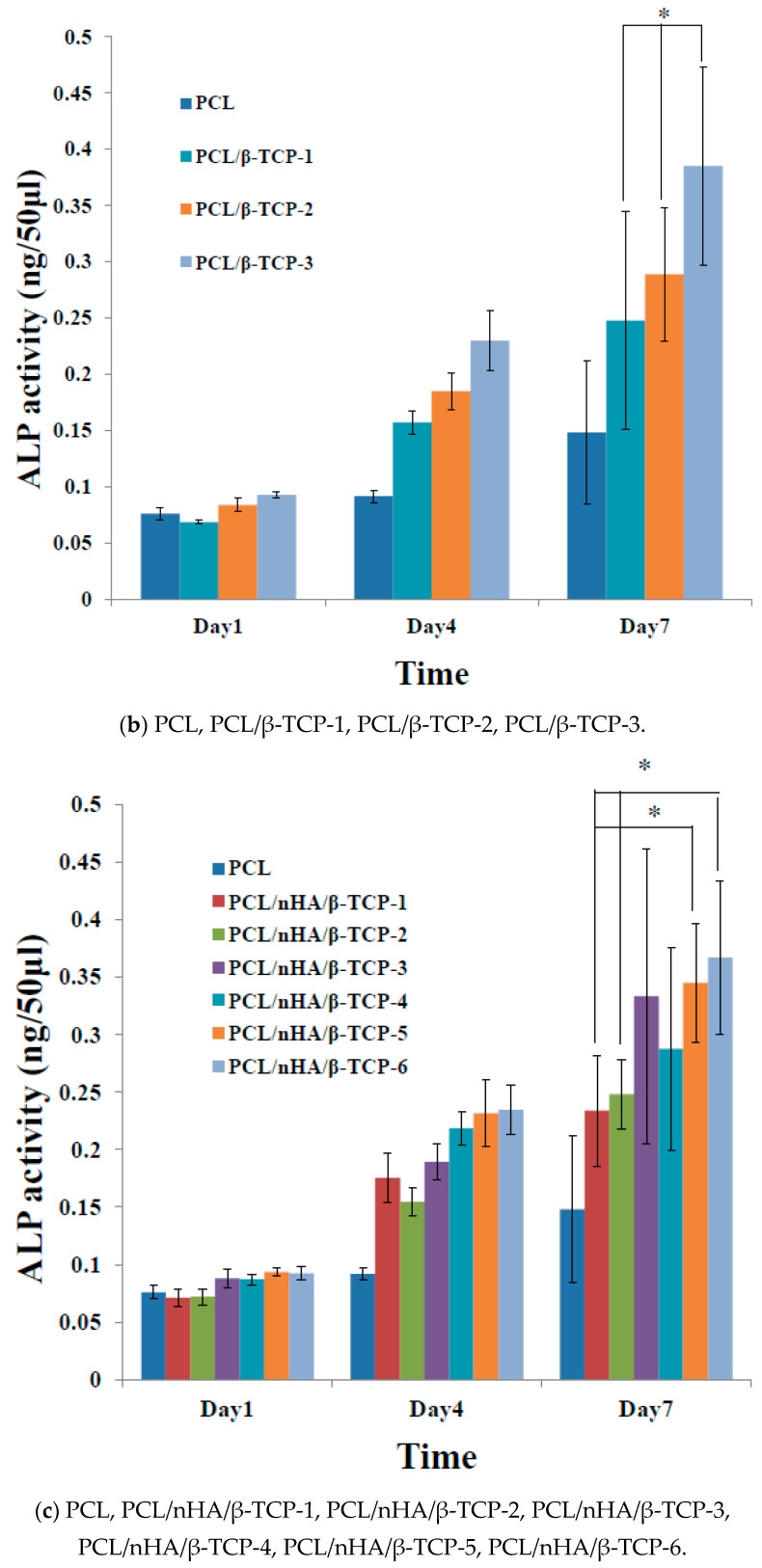
Alkaline phosphatase (ALP) activities of MG63 cells in poly-ε-caprolactone (PCL), PCL/nHA, PCL/β-TCP, and PCL/nHA/β-TCP scaffold samples. (* *p* < 0.05).

Figure 6 shows the proliferation of MG63 osteoblast-like cells cultured in PCL, PCL/nHA, PCL/β-TCP, and PCL/nHA/β-TCP scaffolds, which was measured by ALP activity. Cell proliferation increased as the cell culture time increased. After the first day of cell culture, the scaffolds showed no significant differences. After 7 days of cell culture, ALP concentrations of scaffolds with large amounts of nHA or β-TCP powder were higher Therefore, NPs had an important role in supporting cell proliferation in these scaffolds [25,26]. This study indicated that both nHA and β-TCP contributed to cell growth. Cell attachment and proliferative behavior of the scaffolds depend on their biocompatibility. According to the ALP activity, the biocompatibilities of the scaffolds were in the following order: PCL/nHA-3, PCL/β-TCP-3, PCL/nHA/β-TCP-6 > other scaffolds. The following is a separate comparison of ALP activities of the 3D composite scaffolds containing nHA, /β-TCP, nHA/β-TCP. The rank of ALP activity values was PCL/nHA-3 > PCL/nHA-2 > PCL/nHA-1. The PCL/nHA-3 scaffold had the biggest OD value. The rank of ALP activity values was PCL/β-TCP-3 > PCL/β-TCP-2 > PCL/β-TCP-1. The PCL/β-TCP-3 scaffold had the largest OD value. The rank of ALP activity values was PCL/nHA/β-TCP-6 > PCL/nHA/β-TCP-5 > PCL/nHA/β-TCP-3 > PCL/nHA/β-TCP-4 > PCL/nHA/β-TCP-2 > PCL/nHA/β-TCP-1. The largest ALP activity was for the PCL/nHA/β-TCP-6 scaffold. Cell culture results revealed that the PCL/nHA and PCL/β-TCP scaffolds were suitable for culturing MG63 osteoblast-like cells [63,64].

**Figure 7:**
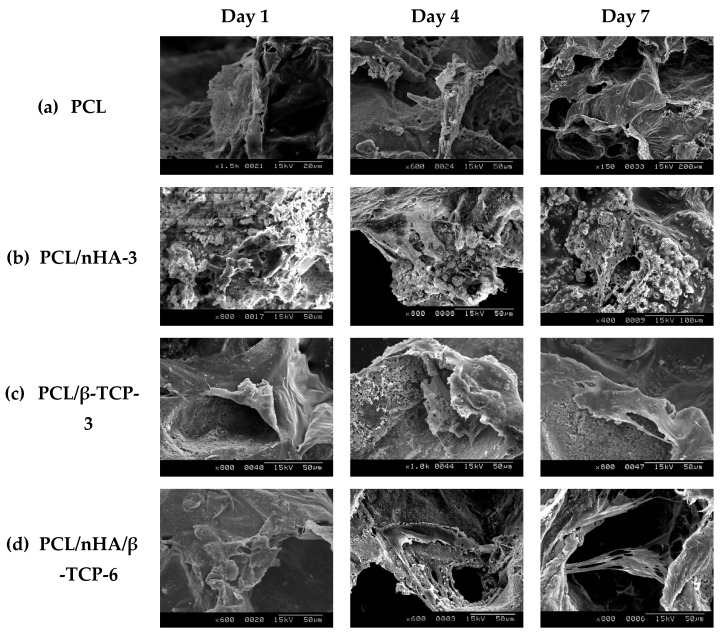
SEM images of the various scaffolds after cell culture: (**a**) poly-ε-caprolactone (PCL), (**b**) PCL/nHA-3, (**c**) PCL/β-TCP-3, and (**d**) PCL/nHA/β-TCP-6.

Figure 7 shows the evolution of the morphology of MG-63 osteoblast-like cells (SEM images) cultured in PCL, PCL/nHA-3, PCL/β-TCP-3, and PCL/nHA/β-TCP-6 scaffolds for 1, 4, and 7 days. Cells had adhered after 1 day. It was noted that cells presented a round morphology. After 4 days of cell culture, cell filopodia had combined together as shown on SEM images. Cells had proliferated in pores of the scaffolds. Results indicated that the scaffolds were suitable for cell attachment. After 7 days, MG-63 osteoblast-like cells were spread throughout the PCL scaffolds. The study results also revealed that cells had formed a cell layer in the PCL/nHA-3, PCL/β-TCP-3, and PCL/nHA/β-TCP-6 scaffolds. The PCL/β-TCP-3 and PCL/nHA/β-TCP-6 scaffolds were full of layer upon layer of cells.

## 4. Conclusions

This study successfully used an improved solvent-casting/particulate-leaching method to fabricate PCL, PCL/nHA, PCL/β-TCP, and PCL/nHA/β-TCP porous scaffolds. All scaffolds had well-interconnected structures and their pore sizes were in the range 250~400 μm. PCL/nHA, PCL/β-TCP, and PCL/nHA/β-TCP scaffolds had greater porosities (>80%) in this study. nHA and β-TCP powders were exposed and evenly dispersed on the scaffold surfaces. The elastic modulus of different composite scaffolds (0.15~1.865 GPa) matched the elastic modulus of human cancellous bone (0.1~4.5 GPa). After mixing in nHA or β-TCP powder, the surface properties of the scaffolds changed from hydrophobic to hydrophilic. Weight loss clearly revealed accelerated degradation of the composite scaffolds. pH values of the composite scaffolds remained in the range 6.25~7.0 after 15 weeks. In vitro cell culture revealed that osteoblast-like MG-63 cells exhibited good attachment (according to an MTT assay) and proliferation (according to an ALP assay) on these composite scaffolds. The MTT test and ALP activity also confirmed that the PCL/nHA, PCL/β-TCP, and PCL/nHA/β-TCP scaffolds with a high ratio of ceramic particles had good in vitro biocompatibility. The rank of ALP activity revealed that the PCL/β-TCP-3 = PCL/nHA/β-TCP-6 > PCL/nHA-3. Therefore, these 3D composite scaffolds were assessed to be suitable for repairing damaged bone by stimulating bioactivity and bone cell formation. This study had limitations in the preclinical biological characterization. In the future, studies should emphasize ectopic examinations of biocompatibility in subcutaneous tissues (to evaluate inflammatory and repair patterns) and orthotopic studies in bone of experimental animals (to evaluate the osseoconductivity and osteoinductive potential). Furthermore, animal experiments with rabbits or dogs could be used, and the results would be more accurate.

## Figures and Tables

**Table 1:** Preparation and characteristics of various scaffolds.

Scaffold	Matrix Material (wt.%)			Porogen (wt.%)		Porosity (%)	Elastic Modulus (GPa)	Contact Angle (°)
	PCL	nHA	β-TCP	(NH)_4_HCO_3_	NaCl			
PCL	30	0	0	20	50	88.10	0.120	70.1
PCL/nHA-1	20	10	0	20	50	87.50	1.005	56.4
PCL/nHA-2	18	12	0	20	50	85.42	1.010	49.9
PCL/nHA-3	15	15	0	20	50	83.20	0.401	45.4
PCL/β-TCP-1	20	0	10	20	50	87.32	0.435	59.2
PCL/β-TCP-2	18	0	12	20	50	84.51	0.381	50.8
PCL/β-TCP-3	15	0	15	20	50	79.35	0.150	46.4
PCL/nHA/β-TCP-1	20	5	5	20	50	85.50	0.479	57.3
PCL/nHA/β-TCP-2	20	6	4	20	50	85.15	1.865	56.9
PCL/nHA/β-TCP-3	18	6	6	20	50	82.51	0.612	50.5
PCL/nHA/β-TCP-4	18	7.2	4.8	20	50	84.23	1.088	49.6
PCL/nHA/β-TCP-5	15	7.5	7.5	20	50	80.35	0.659	46.2
PCL/nHA/β-TCP-6	15	9	6	20	50	81.26	0.826	46.1

## Data Availability

All data used to support the findings of this study are included within the article.

## References

[1] Thomson R.C., Wake M.C., Yaszemsk M.J., Mikos A.G. (1995). Biodegradable polymer scaffolds to regenerate organs. Biopolymers II.

[2] Mikos A.G., Thorsen A.J., Czerwonka L.A., Bao Y., Langer R. (1994). Preparation and characterization of poly(l-lactic acid) foams. Polymer.

[3] Yu H., Matthew H.M., Wooley P.H., Yang S.Y. (2008). Effect of porosity and pore size on microstructures and mechanical properties of poly-ε-caprolactone-hydroxyapatite composites. J. Biomed. Mater. Res. Part B Appl. Biomater..

[4] Wu H., Wan Y., Cao X.Y., Dalai X.Y., Wang S., Zhang S.M. (2008). Fabrication of chitosan-g-polycaprolactone copolymer scaffolds with gradient porous microstructures. Mater. Lett..

[5] Fabbri P., Bondioli F., Messori M., Bartoli C., Dinucci D., Chiellini F. (2010). Porous scaffolds of polycaprolactone reinforced with in situ generated hydroxyapatite for bone tissue engineering. J. Mater. Sci. Mater. Med..

[6] Shaw S.Y., Wu J.Y. (2003). Tissue Engineering of Bone: PLGA-Based Materials as Scaffold. Master’s Thesis.

[7] Causa F., Netti P.A., Ambrosio L., Ciapetti G., Baldini N., Pagani S., Martini D., Giunti A. (2006). Poly-ϵ-caprolactone/hydroxyapatite composites for bone regeneration: In vitro characterization and human osteoblast response. J. Biomed. Mater. Res. Part A.

[8] Shor L., Guceri S., Wen X., Gandhi M., Sun W. (2007). Fabrication of three-dimensional polycaprolactone/hydroxyapatite tissue scaffolds and osteoblast-scaffold interactions in vitro. Biomaterials.

[9] Johari N., Fathi N.H., Golozar M.A. (2012). Fabrication, characterization and evaluation of the mechanical properties of poly (ɛ-caprolactone)/nano-fluoridated hydroxyapatite scaffold for bone tissue engineering. Compos. Part B.

[10] Wan Y., Wu H., Cao X.Y., Dalai S.Q. (2008). Compressive mechanical properties and biodegradability of porous poly (caprolactone)/chitosan scaffolds. Polym. Degrad. Stab..

[11] Ang K.C., Leong K.F., Chua C.K., Chandrasekaran M. (2007). Compressive properties and degradability of poly (ε-caprolatone)/hydroxyapatite composites under accelerated hydrolytic degradation. J. Biomed. Mater. Res. Part A.

[12] Oh S.H., Park I.K., Kim J.M., Lee J.H. (2007). In vitro and in vivo characteristics of PCL scaffolds with pore size gradient fabricated by a centrifugation method. Biomaterials.

[13] Yang S., Leong K.F., Du Z.H., Chua C.K. (2001). The design of scaffolds for use in tissue engineering. Part I. Tradit. factors. Tissue Eng..

[14] Kim S.E., Yun H.S., Hyun Y.T., Shin J.W., Song J.J. (2009). Nano-hydroxyapatite/poly ϵ-caprolactone composite 3D scaffolds for mastoid obliteration. J. Phys. Conf. Ser..

[15] Salerno A., Zeppetelli S., Maio E.D., Iannace S., Netti P.A. (2011). Processing/structure/property relationship of multi-scaled PCL and PCL–HA composite scaffolds prepared via gas foaming and NaCl reverse templating. Biotechnol. Bioeng..

[16] Salerno A., Domingo C. (2015). Pore structure properties of scaffolds constituted by aggregated microparticles of PCL and PCL-HA processed by phase separation. J. Porous Mater..

[17] Jing X., Mi H.Y., Turng L.S. (2017). Comparison between PCL/hydroxyapatite (HA) and PCL/halloysite nanotube (HNT) composite scaffolds prepared by co-extrusion and gas foaming. Mater. Sci. Eng. C.

[18] Petit C., Tulliani J.M., Tadier S., Meille S., Chevalier J., Palmero P. (2019). Novel calcium phosphate/PCL graded samples: Design and development in view of biomedical applications. Mater. Sci. Eng. C.

[19] Maria A.B., Giovanna G.A., Mario M., Paola L., Jean C., Benjamin N., Fulvio D.R., Adriana O. (2015). Functionalized PCL/HA nanocomposites as microporous membranes for bone regeneration. Mater. Sci. Eng. C.

[20] Alessandra T., Aurelio S., Giorgia I., Concepción D., Francesco U., Paolo A.N. (2017). PCL–HA microscaffolds for in vitro modular bone tissue engineering. J. Tissue Eng. Regen. Med..

[21] Suffiyana A., Istikamah S.R., Wati S., Muhammad H.I. (2018). Performance of polycaprolactone/hydroxyapatite (PCL/HA) composite blended by ultrasound assisted melt blending. J. Mech. Eng..

[22] Maria G.G., Núria P.G., Ana M.L.P., Concepción D., Leticia H.R. (2020). Multi-layered polydopamine coatings for the immobilization of growth factors onto highly-interconnected and bimodal PCL/HA-based scaffolds. Mater. Sci. Eng. C.

[23] Koh Y.H., Jun I.K., Kim H.E. (2006). Fabrication of poly("-caprolactone)/hydroxyapatite scaffold using rapid direct deposition. Mater. Lett..

[24] Jacob M.M., Tao X., Qingqing Y., Fang F., Josh D.C., Zhongkui H., Jianning T., Hao F., Hongli S. (2018). Functionalization of PCL-3D electrospun nanofibrous scaffolds for improved BMP2-induced bone formation. Appl. Mater. Today.

[25] Shadi H., Ali K.P., Ahmad O., Tahereh T.K. (2019). Preparation and characterization of PLA/PCL/HA composite scaffolds using indirect 3D printing for bone tissue engineering. Mater. Sci. Eng. C.

[26] Tian L., Zhang Z., Tian B., Zhang X., Wnag N. (2020). Study on antibacterial properties and cytocompatibility of EPL coated 3D printed PCL/HA composite scaffolds. RSC Adv..

[27] Lilian S., Nilza R., Maria B.A.P., Liliana G., Cassilda C.R., Eliandra S.T., Maria H.F., Susana R.S., Fernando J.M. (2019). Influence of PLLA/PCL/HA scaffold fiber orientation on mechanical properties and osteoblast behavior. Materials.

[28] Jang H.Y., Shin J.Y., Oh S.H., Byun J.H., Lee J.H. (2020). PCL/HA hybrid microspheres for effective osteogenic differentiation and bone regeneration. ACS Biomater. Sci. Eng..

[29] Liu L., Wang Y.Y., Guo S.G., Wang Z.Y., Wang W. (2012). Porous polycaprolactone/nanohydroxyapatite tissue engineering scaffolds fabricated by combining NaCl and PEG as co-porogens: Structure, property, and chondrocyte–scaffold interaction in vitro. J. Biomed. Mater. Res. Part B.

[30] Koupaei N., Karkhaneh A. (2016). Porous crosslinked polycaprolactone hydroxyapatite networks for bone tissue engineering. Tissue Eng. Regen. Med..

[31] Rezaei A., Mohammadi M.R. (2013). In vitro study of hydroxyapatite/polycaprolactone (HA/PCL) nanocomposite synthesized by an in situ sol–gel process. Mater. Sci. Eng. C.

[32] Yin H.M., Qian Y.J., Zhang J., Lin Z.F., Li J.S., Xu J.Z., Li Z.M. (2016). Engineering porous poly(lactic acid) scaffolds with high mechanical performance via a solid state extrusion/porogen leaching approach. Polymers.

[33] Kim J.W., Shin K.H., Koh Y.H., Hah M.J., Moon J.Y., Kim H.E. (2017). Production of poly(ε-caprolactone)/hydroxyapatite composite scaffolds with a tailored macro/micro-porous structure, high mechanical properties, and excellent bioactivity. Materials.

[34] Moeini S., Mohammadi M.R., Simchi A. (2017). In-situ solvothermal processing of polycaprolactone/hydroxyapatite nanocomposites with enhanced mechanical and biological performance for bone tissue engineering. Bioact. Mater..

[35] Sari M., Hening P., Chotimah Ana I.D., Yusuf Y. (2021). Bioceramic hydroxyapatite-based scaffold with a porous structure using honeycomb as a natural polymeric porogen for bone tissue engineering. Biomater. Res..

[36] ShiraliPour F., Shafiei S.S., Nikakhtar Y. (2021). Three-dimensional porous poly(ε-caprolactone)/beta-tricalcium phosphate microsphere-aggregated scaffold for bone tissue engineering. International J. Appl. Ceram. Technol..

[37] Susmita B., Naboneeta S., Sahar V. (2019). Sustained release of vitamin C from PCL coated TCP induces proliferation and differentiation of osteoblast cells and suppresses osteosarcoma cell growth. Mater. Sci. Eng. C.

[38] Ethan N., Alexandra R., Amir D., Warren L.G. (2017). Comparison of 3D-printed poly-e-caprolactone scaffolds functionalized with tricalcium phosphate, hydroxyapatite, bio-oss, or decellularized bone matrix. Tissue Eng. Part A.

[39] Choy D.K., Seng N., Nga V.D.W., Lim J., Lu J., Chou N., Yeo T.T., Teoh S.H. (2013). Brain tissue interaction with three-dimensional, honeycomb polycaprolactone-based scaffolds designed for cranial reconstruction following traumatic brain injury. Tissue Eng. Part A.

[40] Lee S., Choi D., Shim J.H., Nam W. (2020). Efficacy of three-dimensionally printed polycaprolactone/beta tricalcium phosphate scafold on mandibular reconstruction. Sci. Rep..

[41] Sawyer A.A., Song S.J., Susanto E., Chuan P., Lam C.X.F., Woodruff M.A., Hutmacher D.W., Cool S.M. (2009). The stimulation of healing within a rat calvarial defect by mPCL–TCP/collagen scaffolds loaded with rhBMP-2. Biomaterials.

[42] Yeo M.G., Kim G.H. (2012). Preparation and characterization of 3D composite scaffolds based on rapid-prototyped PCL/β-TCP struts and electrospun PCL coated with collagen and HA for bone regeneration. Chem. Mater..

[43] Berner A., Woodruff M.A., Lam C.X.F., Arafat M.T., Saifzadeh S., Steck R., Ren J., Nerlich M., Ekaputra A.K., Gibson I. (2014). Effects of scaffold architecture on cranial bone healing. Int. Assoc. Oral Maxillofac. Surg..

[44] Hu Y.Y., Wang J., Xing W., Cao L., Liu C.S. (2014). Surface-Modified Pliable PDLLA/PCL/b-TCP Scaffolds as a Promising Delivery System for Bone Regeneration. J. Appl. Polym. Sci..

[45] Bae E.B., Park K.H., Shim J.H., Chung H.Y., Choi J.W., Lee J.J., Kim C.H., Jeon H.J., Kang S.S., Huh J.B. (2018). Efficacy of rhBMP-2 loaded PCL/b-TCP/bdECM scaffold fabricated by 3D printing technology on bone regeneration. BioMed Res. Int..

[46] Ezati M., Safavipour H., Houshmand B., Faghihi S. (2018). Development of a PCL/gelatin/chitosan/β-TCP electrospun composite for guided bone regeneration. Prog. Biomater..

[47] Alehosseini M., Golafshan N., Kharaziha M., Fathi M., Edris H. (2018). Hemocompatible and bioactive heparin-loaded PCL-α-TCP fibrous membranes for bone tissue engineering. Macromol. Biosci..

[48] Bruyas A., Lou F., Alexander M.S., Michael G., William M., Stuart G., Yang Y.P. (2018). Systematic characterization of 3D-printed PCL/β-TCP scaffolds for biomedical devices and bone tissue engineering: Influence of composition and porosity. J. Mater. Res..

[49] Kang J.H., Kaneda J., Jang J.G., Sakthiabirami K., Lui E., Kim C., Wang A., Park S.W., Yang Y.P. (2020). The influence of electron beam sterilization on in vivo degradation of β-TCP/PCL of different composite ratios for bone tissue engineering. Micromachines.

[50] Araujo L.K., Antunes G.S., Melo M.M., Castro S. (2020). Brazilian dentists′ perceptions of using bone grafts: An inland survey. Acta Odontol Latinoam..

[51] Chen S.Y., Jang T.S., Pan H.M., Jung H.D., Sia M.W., Xie S.Y., Hang Y., Mark S.K., Wang D.G., Song J. (2020). 3D freeform printing of nanocomposite hydrogels through in situ precipitation in reactive viscous fluid. Int. J. Bioprint..

[52] Yang J., Shi G.X., Bei J.H., Wang S.G., Cao Y.L., Shang Q.X., Yang G.H., Wang W.J. (2002). Fabrication and surface modification of macroporous poly(L-lactic acid) and poly(L-lactic-co-glycolic acid) (70/30) cell scaffolds for human skin fibroblast cell culture. J. Biomed. Mater. Res..

[53] Heo S.J., Kim S.E., Wei J., Hyun Y.T., Yun H.S., Kim D.H., Shin J.W. (2009). Fabrication and characterization of novel nano-and micro-HA/PCL composite scaffolds using a modified rapid prototyping process. J. Biomed. Mater. Res..

[54] Ramires P.A., Romito A., Cosentino F., Milella E. (2001). The influence of titania/hydroxyapatite composite coatings on in vitro osteoblasts behavior. Biomaterials.

[55] Smejkal G.B., Kaul C.A. (2001). Stability of Nitroblue Tetrazolium-based Alkaline Phosphatase Substrates. J. Histochem. Cytochem..

[56] Lien S.M., Ko L.Y., Huang T.J. (2009). Effect of pore size on ECM secretion and cell growth in gelatin scaffold for articular cartilage tissue engineering. Acta Biomater..

[57] Wang Y., Dai J., Zhang Q.C., Xiao Y., Lang M. (2010). Improved mechanical properties of hydroxyapatite/poly (ɛ-caprolactone) scaffolds by surface modification of hydroxyapatite. Appl. Surf. Sci..

[58] Alessandro P., Dario P., Manuela P., Rodoffo Q., Silvia S. (2011). Osteoinduction of human mesenchymal stem cells by bioactive composite scaffolds without supplemental osteogenic growth factors. PLoS ONE.

[59] Wang J., Zhou B., Liu X.S., Fields A.J., Sanyai A., Shi X.T., Adams M., Keaveny T.M., Guo X.E. (2015). Trabecular plates and rods determine elastic modulus and yield strength of human trabecular bone. Bone.

[60] Morgan E.F., Bayraktar H.H., Keaveny T.M. (2003). Trabecular bone modulus–density relationships depend on anatomic site. J. Biomech..

[61] Loher S., Reboul V., Brunner T.J., Simonet M., Dora C. (2006). Improved degradation and bioactivity of amorphous aerosol derived tricalcium phosphate nanoparticles in poly (lactide-co-glycolide). Nanotechnology.

[62] Vollenweider M., Brunner T.J., Knecht S., Grass R.N., Zehnder M., Imfeld T., Stark W.J. (2007). Remineralization of human dentin using ultrafine bioactive glass particles. Acta Biomater..

[63] Lomelino R.O., Castro-Silva I., Linhares A.B.R., Alves G.G., Santos S.R.A., Gameiro V.S., Rossi A.M., Granjeiro J.M. (2012). The association of human primary bone cells with biphasic calcium phosphate (bTCP/HA 70:30) granules increases bone repair. J. Mater. Sci. Mater. Med..

[64] Castro-Silva I.I., Zambuzzi W.F., Castro L.O., Granjeiro J.M. (2012). Periosteal-derived cells for bone bioengineering: A promising candidate. Clin. Oral Implant. Res..

